# Beyond Transition: A Substantive Grounded Theory of Forward Escaping Among Newly Qualified Nurses

**DOI:** 10.1155/jonm/3450332

**Published:** 2026-08-16

**Authors:** Nasser Aldosari, Steven Pryjmachuk, Hannah Cooke

**Affiliations:** ^1^ Division of Nursing, Midwifery and Social Work, School of Health Sciences, University of Manchester, Manchester M13 9PL, UK, manchester.ac.uk; ^2^ Research Center, King Abdullah Medical City, Mecca 24246, Saudi Arabia, kamc.med.sa

**Keywords:** grounded theory, newly qualified nurses, nursing management, transition to practice, workforce retention

## Abstract

**Background:**

Early‐career nurse attrition is recognised as a critical nursing management and workforce governance challenge worldwide. Despite investment in structured transition programmes, alarming retention rates amongst newly qualified nurses persist, particularly in contexts where nursing traditionally occupies a low or ambiguous social position. Evidence informing management and policy responses to early‐career mobility in such settings is limited.

**Methods:**

A classic grounded theory design explored transition experiences and their nursing management implications among newly qualified nurses in Western Saudi Arabia. Data were collected through conversational, in‐person interviews with 33 participants, 19 newly qualified nurses and 14 key informants, across two institutions, supplemented by document analysis. Data were analysed using constant comparative analysis following the classic grounded theory tradition of Glaser and Strauss to guide theory development.

**Results:**

Analysis generated nine categories integrated into three subcore categories, Acquiescing, Reconcile and Perseverance, and one core category, Forward Escaping. This theory conceptualises newly qualified nurses remaining in the profession while strategically orienting towards roles offering greater autonomy, social legitimacy and advancement. Departure from bedside nursing reflected ambivalence shaped by social stigma, limited role clarity and constrained career pathways, rather than professional disengagement. Saudi Arabia’s Vision 2030 reform agenda, expanding advanced practice visibility and leadership pipelines, further shaped nurses’ workforce orientations, offering lessons for other reform‐driven health systems.

**Conclusion:**

The Forward Escaping substantive theory challenges management strategies that rely predominantly on career progression as a retention lever, highlighting the risk of neglecting persistent bedside conditions. Sustainable workforce retention requires nursing leadership to integrate career mobility structures with active investment in bedside role quality, recognition and working conditions.

**Implications for Nursing Management:**

Nurse managers and leaders must move beyond advancement‐focused retention models to address professional recognition and role clarity at the bedside. Governance frameworks should align workforce policy with healthcare reform agendas to elevate nursing’s social standing, strengthen leadership pipelines and embed evidence‐based management practices that sustain newly qualified nurses where patient care demand is greatest.

## 1. Background

Shortage of registered nurses has been called a worldwide health emergency [1]. The World Health Organization [2] (WHO) has stated that a worldwide shortage of nearly 4.5 million nurses is predicted for 2030. This shortage will most likely compromise how care is administered, which will likely lead to a greater number of deaths for patients [3].

In response to this shortfall, numerous nations are concentrating on the supply pipeline, i.e., the expansion of nurse student allocations and/or the establishment of additional nursing institutions [4]. It will not matter if newly qualified nurses (NQNs) leave the profession when incorporating pipeline strategies. As stated by Leary [5], retention is ‘the hole in the leaky workforce bucket’. The NQNs exhibit alarming attrition rates (as high as 25%), which endangers the sustainability of the workforce within the profession [6]. In certain cases, some pipeline tactics could prove to be detrimental, such as Salamonson et al. [7] reported that selecting nursing as a first‐choice degree was a strong predictor of completion of the program. Recruiting a high proportion of second‐choice students was thus linked with a higher rate of attrition.

With the risk of attrition being quite significant, moving from being a student to a qualified nurse becomes an especially important and sensitive time period. The emotional and professional challenges of being a new nurse, which lead to early attrition, are partly explained by Kramer’s ‘reality shock’ theory and Duchscher’s ‘stage of transition’ theory [8, 9]. Numerous countries have implemented support programmes such as preceptorships and residency programmes to alleviate early attrition. However, the effectiveness of these approaches remains inconclusive [10].

In Saudi Arabia, these problems are occurring in a context of fast societal change. Since the profession’s inception, there has been social stigma regarding bedside care and involvement in co‐ed settings [11]. Since the introduction of Saudi Vision 2030 in 2016, policy reforms have improved public perceptions of healthcare professions through workforce empowerment, normalisation of mixed‐gender workplaces and increased visibility of health professionals [12].

As a result of the aforementioned changes, the health system in the area still depends significantly on foreign nurses, making up more than half of the staff (56%) [13]. In the context of the broader Saudization policies aimed at modernising the economy and building up the national workforce, the universities and the policymakers are increasingly focused on recruiting Saudi nationals to nursing programs [14]. Empirically grounded understanding of Saudi nurses’ transition experiences amid ongoing sociocultural change remains limited. The current body of work analysed in the studies has been predominantly focused on barriers and challenges, while less attention has been given to the possible motivations, changes in coping strategies and career pathways that might evolve in response to the changes.

This research covers this void by focusing on experiences of NQNs regarding their transition into professional nursing practice and how sociocultural transition, professional role formation and goals of career mobility influence retention and sustainability of the workforce.

## 2. Methods

### 2.1. Study Aim

The purpose of this study is to explore the transition experiences of NQNs into professional nursing practice.

### 2.2. Study Design

A classic grounded theory (CGT) approach was used to explore the transition experiences of NQNs. CGT is particularly suited to investigating underexplored social processes, as it enables theory generation grounded in participants’ experiences and moving up from a descriptive to conceptual level of understanding a phenomenon. It involves simultaneous data collection and analysis, constant comparative methods and the development of a substantive theory that explains participants’ main concerns and how these are resolved over time. Although more recent grounded theory approaches provide alternative procedural and epistemological pathways, this study adopted the classic Glaserian approach, emphasising the inductive emergence of theory from the data rather than the application of a predetermined analytic framework [15]. The study adhered to the Standards for Reporting Qualitative Research (SRQR) checklist to enhance transparency, rigour and completeness in study design, conduct and reporting.

### 2.3. Setting and Access

The study participants were selected from one university and one quaternary hospital situated in Western Saudi Arabia. The latter had started a nursing residency programme (NRP) and was looking to recruit and retain NQNs. The two institutions’ central nursing departments aided in recruiting candidates but were not informed regarding the participants’ recruitment decisions to maintain confidentiality and minimise coercion. 33 individuals were interviewed, of whom 19 were NQNs, and 14 were supportive professionals of the NQNs, including preceptors, clinical resource nurses and nurse managers (NM). The first round of purposive sampling comprised nine NQNs in the first year of their clinical practice to discuss and gain insight into the transitional experience. The sampling then moved to theoretical sampling; 10 additional NQNs were included based on the emerging concepts of professional autonomy, acceptance of nursing by the general public and interest in advanced practice.

### 2.4. Sampling

Participant recruitment continued until theoretical saturation was achieved, defined as the point at which additional data yielded no new theoretical insights. To further support data triangulation, the curriculum of the NRP and the student handbook were reviewed. The majority of NQNs were 23–27 years old, female and living with their parents. Each NQN was also completing a 12‐month NRP, which had activities such as clinical placements, shadowing a senior nurse, a series of teaching sessions and ongoing formative and summative evaluations.

### 2.5. Data Collection

Following the principles outlined in CGT, 40–60 min of conversational, in‐person interviews were conducted. Given their investigative aims, the first questions in each interview were relatively general, so participants would feel free to ramble on the issues at hand (e.g., ‘How is work going?’). With the participants’ permission, interviews were recorded, and the researcher supplemented these with field observations and memos to aid the ongoing analytic process and development of theory. To expand and specify the principal categories with respect to the NRP and early‐stage professional role development, the researcher conducted second interviews with four of the participants.

### 2.6. Data Analysis

Interviews were transcribed verbatim and analysed using constant comparative analysis. Data analysis was conducted manually without the use of qualitative data analysis software, consistent with the CGT tradition that emphasizes direct and unmediated researcher engagement with the data [16]. In collecting and analysing the data, each interview was considered individually, and the resulting data guided the sampling and the questions in subsequent interviews. Using open coding, the preliminary data were given initial codes that were constantly compared both within and across interviews and were eventually grouped into various conceptual groups. Throughout the process, memo writing, as well as diagramming, was utilised to outline various decisions made and the interrelations of the categories, as well as to assist in the integration of the theory. Subsequently, the data were structured into codes, subcore categories, and one core category that created the foundation of the emergent substantive theory.

## 3. Results

### 3.1. Overview/Figure 1

**FIGURE 1:**
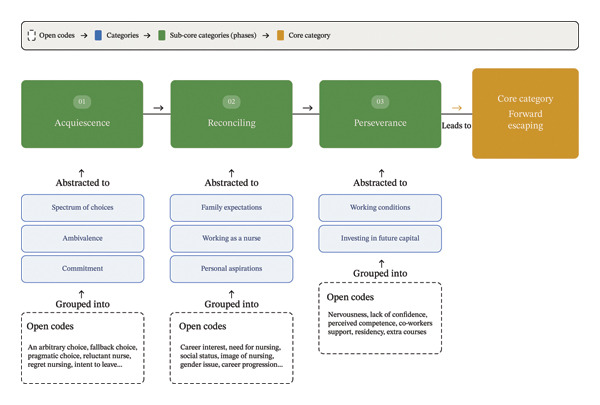
Coding process.

The results of this study are summarised in Figure 1, which displays the analytic progression from the initial codes to the categories, subcore categories and the core category. During the constant comparative analysis, nine categories were formed and later on consolidated into three subcore categories: Acquiescing, Reconciling and Perseverance. These subcore categories were merged in theory with the core category of Forward Escaping, which describes the strategies of NQNs to deal with their primary concern of staying in nursing while managing ambivalence, limited and shifting professional roles. In this regard, Forward Escaping reflects a dual process of distancing from low‐valued bedside roles while pursuing progression towards higher status nursing roles that offer more flexibility and autonomy.

Each subcore category is discussed individually in the section that follows, including a description of the core category.

### 3.2. Subcore Category 1: Acquiescence

This describes how NQNs first accepted nursing as a career. This initial acceptance, often passive acceptance, crafted how NQNs began to adapt to their first role. This subcore category describes how participants felt they had limited agency, ongoing reluctance, efforts to embrace nursing and a hesitance to fully pursue it.

#### 3.2.1. The Spectrum of Choices

University students in Saudi Arabia have to complete a foundation year before they can select a specialisation, with students who wish to study the health professions needing to enter a medical track. Most NQNs described their initial aspirations as being in the medical field due to the societal prestige and expectations from their families, with nursing seen as a secondary option or a fallback. However, recent cohorts have been described as being more aware of nursing as a modern, skilled profession with opportunities for leadership, advanced practice and significant nursing autonomy, and this is changing their perceptions more positively. The awareness and exercise of nursing as a first and only career choice, however, was rare, with only one participant explicitly stating that nursing was their first‐choice career. Most participants described a lack of choice in their decisions, and this appears to have been influenced by a combination of academic results, expectations of those around them, their family and the way in which they were assigned a place to study. Five broad categories of lack of choice were described, which were named arbitrary, fallback, pragmatic, family and coerced. Lack of choice for some participants was due to a lack of awareness of nursing as a profession.

*‘To be honest, I knew nothing about nursing when I first enrolled*… *It was just a random choice [laughs].’ —NQN11*

Most participants believe that public awareness campaigns, school outreach and career guidance efforts improve understanding of nursing roles and scope of practice compared to when they entered the nursing profession a few years ago. Participants described a fallback option when they were unsuccessful in gaining entry to medicine. Assignment to nursing was often received as disappointing and socially devalued, especially when reinforced by grades in the foundation year. During foundation year, nursing was described as closely related to medicine and thus some students were encouraged to select nursing as their second option rather than other allied health professions. Participants indicated a change in faculty messaging to increasing emphasis on nursing leadership and its distinct roles, which has shifted in importance to nursing leadership alignment with modernisation in the country. The emergence of government initiatives to upgrade nursing, its remuneration and advanced practice roles (such as Nurse Practitioner), which afford autonomy, clinical decision‐making and career advancement, prompted some participants to make a ‘pragmatic’ choice.

*‘While I was interested in pharmacology, considering employment opportunities and professional growth, nurses have a better chance compared to pharmacists.’ —NQN5*

Family‐influenced career options were also common, particularly among female participants whose families preferred that they remain close to home. Although cultural requirements still limit options, most participants reported increases in family acceptance and major culture change, especially with mixed‐gender workplaces being more entrenched in Vision 2030.

*‘My family were OK with choosing nursing although it requires working with men. That would have been an issue let’s say 10 years back’ —NQN7*

Participants who were compelled to choose nursing through the pressure of universities because of the programme demand reported a coerced choice.

Conversely, a few of the participants were strategic in their choice and chose nursing despite social opposition. These people were observed to be more confident, better‐planned in their careers and professionally committed, and this has also been noted by preceptors and managers.

*‘In the past, there were more local male nurses, but that started to change in the last 10-15 years.’—NM02*

#### 3.2.2. Ambivalence Towards Nursing

Several NQNs expressed ambivalence and regret regarding their nursing careers and future professional trajectories. Most of this ambivalence belonged to those who felt confined to the nursing profession.

*‘There’s a war inside me: Is nursing for me?’—NQN1*

Except for the most extreme ones, every case involved some form of ambivalence, including those who made a more practical choice while still considering the complexities of the field. Although ambivalence might be more extreme in some cases than in others, all participants in the study experienced ambivalence to some degree. This, in all cases, had a direct effect on the levels of commitment participants had to the field of nursing.

#### 3.2.3. Commitment to the Nursing Profession

NQNs demonstrated varying degrees of commitment to the profession. Some were committed to remaining in nursing, either in their current role or with aspirations for career advancement, viewing nursing as a potential lifelong career.

*‘The team here is fantastic and supportive. You might see me here in the same job when I’m 60 years old.’—NQN10*

People were dedicated to nursing and even involved in considering other career paths that were at a higher rank and had more independence. These individuals viewed nursing as a profession and focused on growing within it. Some, however, were unhappy in their positions and were looking to either exit nursing completely or stop working in bedside care. Still, a lot of these people expressed wanting to stay within the nursing profession and were solely looking for positions for more advanced or specialised roles with more independence.

*‘I will not continue on the bedside… unless they offer us a advanced roles with more professional privileges like independent decision-making’—NQN4*

### 3.3. Subcore Category 2: Reconciling

Reconciling explains how the NQNs were trying to reconcile family expectations and personal ambition with the realities of nursing practice. Public awareness of the professional value of nursing, increased acceptance of working in mixed‐gender settings and the presence of leadership and advanced practice roles continued to influence this process.

#### 3.3.1. Reconciling Family Expectations With Work Demands

The participants reported early disapproval of family members due to the perception of nursing as socially inappropriate. With time, the perspective of many families changed because they had an understanding of the clinical responsibility, expertise and social contribution of being a nurse.

*‘When I explain what we do… they value and appreciate our work.’—NQN2*

*‘Telling them [family] stories about how we save lives positively change their views of nurses and nursing work.’—NQN5*

The fear of mixed‐gender workplaces remained among some of them, especially the female participants, yet the fear was being increasingly redefined as unavoidable in an evolving society.

Interestingly, this cultural rejection of mixed‐gender workplaces did not appear to be the case with high‐status jobs like medicine. As the government continues to drive towards a prosperous and non‐discriminatory workforce, more families are starting to perceive nursing as a valued and respected professional job.

#### 3.3.2. Clinical Practice Experiences of Newly Qualified Nurses

Early clinical experiences sometimes reinforced negative perceptions due to hierarchical dynamics or lack of recognition. However, many participants described growing professional pride, particularly when their expertise was recognised by patients and physicians.

*‘We as ICU nurses are very trusted and respected by the vast majority of physicians including the senior ones.’—NQN13*

Although work overload and night shifts continued to be an issue, NQNs reported employing professional development, mentorship and career planning as the coping mechanisms regarding the issue.

#### 3.3.3. Reconciling Personal Aspirations With a Future Nursing Career

Economic incentives, professional development opportunities and the visibility of nurses in senior leadership roles were described as powerful symbols of possibility.

*‘Seeing nurses as hospital directors sends a message that we are trusted, valued, and the older picture [of nursing] is changing.’—NQN19*

Such changes have helped both nurses and their families overcome outdated perceptions of the profession.

### 3.4. Subcore Category 3: Perseverance

Perseverance reflects how NQNs continued in nursing despite challenging working conditions, performance anxiety and organisational pressures. Perseverance was not passive endurance; it was an active, goal‐directed process through which NQNs sustained engagement with nursing while simultaneously building the work experience necessary to advance beyond current constraints. Two analytically distinct subthemes constituted this category: Tolerating conditions as a transitional strategy and Investing in future capital.

#### 3.4.1. Tolerating Conditions as a Transitional Strategy

NQNs consistently described demanding working conditions, including 12‐h shifts, rotating schedules and high patient acuity, as burdens they consciously accepted as time‐limited features of an early career phase, rather than permanent fixtures.

*‘I keep telling myself this is temporary. Everyone goes through this at the beginning. I just have to get past this stage.’—NQN3*

*‘12-hour shifts cannot fit in nicely with family and social life. They aren’t ideal, especially for married nurses.’—NQN14*

This framing, tolerating present conditions in exchange for future positioning, was a recurring cognitive strategy. NQNs who demonstrated this orientation were distinguished from those who responded to similar conditions with disengagement or exit intention. Preceptors and NMs independently noted that NQNs who displayed perseverance tended to invest more actively in professional development activities and were perceived as more likely to remain in nursing.

#### 3.4.2. Investing in Future Capital

In parallel with tolerating challenging working conditions, NQNs described deliberate efforts to strengthen their professional profiles through training, formal coursework, peer networking and mentorship engagement.

*‘I am taking extra online courses on my days off. I know it is tiring, but I want to improve my skills in different areas like research, quality and leadership.’—NQN8*

*‘I attend every workshop the residency program offers… even elective ones. I want my CV to be ready.’—NQN12*

This active investment in future‐oriented professional capital is central to understanding Perseverance as a strategic process. NQNs were not simply surviving current conditions; they were accruing the qualifications, experiences and professional relationships that would enable eventual Forward Escaping towards higher status roles. Therefore, Perseverance was the dynamic bridge between early‐career acquiescence and the forward‐oriented mobility captured by the core category.

### 3.5. The Core Category: Forward Escaping

The main category of Forward Escaping includes three subcategories: Acquiescing, Reconciling and Perseverance. NQNs attempt to stay in nursing as a vent for their main concern, obtaining social legitimacy, independence and advancement. Forward Escaping began with a discomfort with nursing, dissatisfaction with nursing as a low‐status profession and ignorance of the wide variety of nursing careers. However, with the Vision 2030 reforms, these views have changed. There is now more respect for nursing, mixed‐gender workplaces are common, and advanced nursing practice is more visible and attainable. In this new context, Forward Escaping signifies a nursing role, but the goal is to use that role as a stepping stone for upward movement in the field. For many NQNs, the goal is not to leave nursing but to advance within the profession to achieve independence, decision‐making authority and social recognition. Given the opportunity, Forward Escaping will most likely transform from an indication of the risks of nursing retention to a means of promoting a steady nursing workforce and retaining passionate and talented nurses in the profession.

## 4. Discussion

This study examines why NQNs attempt to transition away from bedside roles, conceptualised as Forward Escaping. The findings indicate that NQNs are not disengaging from nursing but are seeking advanced roles and professional autonomy. This pattern resonates with international evidence on early‐career nurse mobility and professional identity formation, while simultaneously reflecting the influence of Saudi Arabia’s Vision 2030 reform agenda. Rather than representing a departure from global trends, Forward Escaping demonstrates how macro‐level policy reform, in this case, the rapid expansion of advanced practice visibility, women’s workforce participation and efforts to clarify the authentic image of the nursing profession, can actively reconfigure the career orientations and retention dynamics of a newly qualified nursing workforce. These findings carry implications not only for Saudi nursing management but also for any health system undergoing accelerated sociocultural and policy transformation.

### 4.1. Vision 2030 and the Transformation of Nursing Identity

In Saudi Arabia, the nursing profession has historically been constrained by perceptions of low social status, gendered expectations and limited professional autonomy [17]. Vision 2030 represents a major structural change, prioritising the establishment of a prosperous community, increasing women’s participation in the workforce, normalising co‐ed work environments and facilitating higher and more complex roles in nursing. These changes, as a whole, are starting to change the professional identity and the social recognition of nursing. Other countries experiencing similar changes at a national scale demonstrate that changes in macro policy can directly impact professional identity, involvement and retention. For example, in the United Arab Emirates, national nurse retention has improved with the introduction of structured career pathways under the Emiratization policy [18]. Similarly, in the United Kingdom, sustained national investment in nurse education, role diversification and advanced practice pathways has been linked to higher professional engagement and longer career trajectories [6].

However, some authors caution against assuming that policy rhetoric alone translates into sustained workforce change. Evidence from health workforce reforms internationally, including workforce nationalisation initiatives in Gulf countries such as Oman, suggests that policy‐driven professionalisation can raise expectations faster than organisational capacity can absorb them [19, 20]. When expanded career opportunities, authority or professional recognition fail to materialise in practice, disillusionment and attrition may follow. This raises the question of whether Forward Escaping represents a sustainable retention mechanism or merely a delayed attrition pathway if institutional reforms fail to keep pace with evolving professional expectations.

### 4.2. Advanced Practice Roles as a Global Retention Lever

In this study, the ambition to take on an advanced role such as a nurse practitioner was delineated as a strong ‘pull factor’. There is global evidence that retention is related to having a higher level of professional autonomy and advancement in one’s career [21, 22]. There is data from the Organisation for Economic Co‐operation and Development (OECD) that shows that allowing nurse practitioners in the full scope of their practice to work increases job satisfaction and stability within the workforce [23].

Studies from across the world identify the same phenomenon: heavy trajectories of nurses′ training and career advancement capture a very small subset of the motivated nurses, while systemic dissatisfaction is overlooked and continues to erode the bedside level of the system. Studies in Canada and Australia, for example, show a disparity in the contentment of the nurse practitioners and the bedside nurses, where the latter complain of professional stagnation, role constriction, worsened workflow and a heavy caseload as senior nurses and staff progressively leave their positions in direct patient care [24, 25]. Within this frame, Forward Escaping runs the risk of embodying a tiered retention strategy, favouring individuals who possess the ability to ‘escape forward’ while leaving underlying structural issues to fester for the remainder. Recent qualitative evidence from Saudi Arabia also suggests that while advanced practice roles exhibit a profound professional identity and remain hopeful, they face challenges such as regulatory uncertainty, payment inconsistencies and a lack of control over leadership positions [26].

### 4.3. Work–Life Integration and Cultural Change

Although NQNs report working conditions that come with 12 h shifts as temporary sacrifices, these characteristics are also found in international transition studies looking at early career nurses who put up with suboptimal conditions in hopes of future advancement. However, evidence from the world increasingly puts this model in question as it becomes apparent that the trade‐off linked with long hours, working shifts, work–life imbalance and increased attrition results in burnout and increased safety risks for patients [27, 28].

In the Saudi context, cultural changes driven by Vision 2030 appear to mitigate family resistance, especially with regard to women working in mixed‐gender settings. Other rapidly modernising societies have shown similar patterns. In China, historically ingrained professional identity stereotypes among nurses, especially regarding gender, have shifted at a gradual pace, driven by changes in the labour market, rather than within the nursing profession [29]. Turkey’s acceptance of nursing as an occupation has improved, thanks to changing social attitudes; however, still existing social hierarchies and gendered structures within workplaces are issues that need to be addressed [30].

### 4.4. Forward Escaping: Implications for Global Nursing

This study’s key theoretical contribution lies in framing Forward Escaping as a future‐oriented, strategic process rather than a reactive withdrawal, extending classic transition theories [8, 9] by incorporating policy reform and sociocultural change as dynamic forces shaping transition trajectories.

Consequently, the findings are also evidence against the positive, uncritical interpretations regarding the understandings of progression in the professions. Internationally, literature covering progression in careers, and especially the progression of careers within the health professions, warns that when advancement is predominantly viewed as the main vehicle for the attainment of professional legitimacy, inadequate bedside roles are likely to become further devalued, and this devaluation is likely to compound shortages in essential clinical roles [31, 32].

## 5. Strengths and Limitations

A key strength of this study lies in its use of CGT to generate a conceptual explanation of Forward Escaping grounded in the lived experiences of NQNs in Saudi Arabia. Rigour was enhanced through simultaneous data collection and analysis, constant comparison, theoretical sampling and data triangulation, consistent with Glaser’s [16] criteria of fit, relevance, workability and modifiability. Including perspectives from NQNs alongside preceptors, educators and managers strengthened contextual understanding of transition processes. Theoretical saturation was achieved, supporting the credibility of the emergent core category. While context‐specific, the underlying process may offer conceptual transferability to other nursing workforces undergoing rapid social or professional change.

Several limitations should be acknowledged. The insider position of the lead researcher (male) facilitated rapport but may have constrained discussion of sensitive issues, particularly those related to gender and power, despite reflexive strategies to mitigate bias. The study’s single‐country focus limits direct transferability, as the influence of Vision 2030 and Saudization policies is contextually unique. Additionally, the absence of perspectives from nurses who had already exited the profession may have limited insight into pathways where Forward Escaping does not resolve core concerns. Future multi‐country and longitudinal research would help clarify how Forward Escaping operates over time as either a retention mechanism or a delayed attrition pathway.

## 6. Conclusions

The substantive theory of Forward Escaping conceptualizes NQNs’ pursuit of professional legitimacy, autonomy and advancement beyond traditional bedside roles. While these ambitions are often considered positively, this pattern exposes a structural tension within contemporary nursing workforce strategies. Advanced roles may improve retention for a select, highly motivated subgroup; however, reliance on progression as a primary retention solution systematically diverts attention from concurrent challenges in bedside nursing, including excessive workload, constrained autonomy and chronic work–life imbalance. In the absence of parallel, substantive investment in the redesign and revaluation of bedside roles, such strategies risk entrenching professional stratification, accelerating burnout and destabilising the core clinical workforce.

By integrating policy reform and sociocultural change into transition theory, this study argues that sustainable retention cannot be achieved through advancement pathways alone. Instead, workforce strategies must explicitly legitimize career mobility while simultaneously restoring the professional value, authority and sustainability of bedside nursing as a central pillar of healthcare delivery.

**Nomenclature**
NQN: Newly qualified nurse
CGT: Classic grounded theory
NM: Nurse manager
HD: Head nurse
WHO: World Health Organization
MOH: Ministry of Health
NRP: Nursing residency programme
OECD: Organisation for Economic Co‐operation and Development

## Author Contributions

Study design: N.A., S.P., H.C.

Data collection: N.A.

Data analysis: N.A., S.P., H.C.

Study supervision: S.P., H.C.

Manuscript writing: N.A.

Critical revisions for important intellectual content: S.P., H.C.

## Funding

This research received no specific grant from any funding agency in the public, commercial or not‐for‐profit sectors.

## Ethics Statement

Ethical approval was secured from both a university (UREC: 2019‐5141‐9284) and a hospital (IRB: 18‐488). Ethical safeguards included informed consent, data governance (confidentiality and anonymity) and participant and researcher safety.

## Consent

The authors have nothing to report.

## Conflicts of Interest

The authors declare no conflicts of interest.

## Data Availability

The datasets used and/or analysed during the current study are available from the corresponding author on reasonable request.
